# Negative or Positive? Loading Area Dependent Correlation Between Friction and Normal Load in Structural Superlubricity

**DOI:** 10.3389/fchem.2021.807630

**Published:** 2022-02-01

**Authors:** Kehan Wang, Jin Wang, Ming Ma

**Affiliations:** ^1^ State Key Laboratory of Tribology, Department of Mechanical Engineering, Tsinghua University, Beijing, China; ^2^ Center for Nano and Micro Mechanics, Tsinghua University, Beijing, China; ^3^ International School for Advanced Studies, Trieste, Italy; ^4^ Institute of Superlubricity Technology, Research Institute of Tsinghua University in Shenzhen, Shenzhen, China

**Keywords:** structural superlubricity, graphene, friction, normal load, molecular dynamics simulation

## Abstract

Structural superlubricity (SSL), a state of ultra-low friction between two solid contacts, is a fascinating phenomenon in modern tribology. With extensive molecular dynamics simulations, for systems showing SSL, here we discover two different dependences between friction and normal load by varying the size of the loading area. The essence behind the observations stems from the coupling between the normal load and the edge effect of SSL systems. Keeping normal load constant, we find that by reducing the loading area, the friction can be reduced by more than 65% compared to the large loading area cases. Based on the discoveries, a theoretical model is proposed to describe the correlation between the size of the loading area and friction. Our results reveal the importance of loading conditions in the friction of systems showing SSL, and provide an effective way to reduce and control friction.

## Introduction

Structural superlubricity (SSL) is a state where the sliding friction approaches to zero due to the cancellation of lateral forces between two solid contacts (Dienwiebel et al., 2004; Hod et al., 2018). The ultra-low friction promises SSL the unprecedented application potential in reducing the industrial energy dissipation and preventing the wear failure of devices like hard drives and micro electro mechanical systems (MEMS) (Kim et al., 2007; Urbakh, 2013; Huang et al., 2021). In practical applications, the extremely low friction coefficient (≤0.001) is considered to be a key characteristic of SSL systems (Martin et al., 1993).

The dependence of friction on normal load, which is usually characterized by the friction coefficient, is a key property of SSL. Regarding this aspect, a few forward-looking simulation studies revealed some interesting phenomena. For example, Mandelli et al. revealed an unexpected negative correlation between friction and the normal load with aligned graphene/hBN heterostructures (Mandelli et al., 2019). Normal load is also found to induce incommensurate-to-commensurate transition on graphitic homogeneous contacts (Wang et al., 2019d). van Wijk et al. observed a sudden and reversible increase in friction with normal loads due to the pinning effect of edge atoms for incommensurately stacked flakes (Van Wijk et al., 2013).

Nevertheless, many phenomena predicted by MD simulations have not been confirmed by experiments so far. Inherent differences between simulations and experiments may lead to the discrepancies, such as differences in size and sliding velocities (Li et al., 2011; Vanossi et al., 2013). However, there is another significant difference between the existing MD simulations and experiments: the size of the loading area. In MD simulations, usually a uniform normal load is applied to all atoms on the contact area (Van Wijk et al., 2013; Wang et al., 2019d; Mandelli et al., 2019). In SSL experiments, atomic force microscope (AFM) is often used to press and drive the graphite island (Song et al., 2018; Liu et al., 2020a; Liao et al., 2021). The curvature radius of the AFM tip is in the order of 10–100 nm, while the side length of the graphite island is in the order of 1 μm (Liu et al., 2012; Vu et al., 2016; Liu et al., 2018a; Song et al., 2018). Recent studies show that the area experiencing prominent normal load only occupies a small part of the entire contact area (Song et al., 2018). Given that AFM is commonly used in SSL experiments, it is of great significance to clarify the effect of loading area on friction.

Here in this work, we investigate the effect of the size of the loading area on the interlayer friction of graphene by MD simulations. We find that friction shows a non-monotonic dependence on the normal load for small loading area cases, while a linear dependence is observed for large loading area cases. Our discoveries can be well explained by the coupling effect between the normal load and the edge dissipation. For the same normal load, we also discover that by reducing the loading area, the friction can be reduced by more than 65% compared to the large loading area cases, providing an effective way to reduce and control friction. Based on these findings, we propose a theoretical model to describe the dependence between the size of the loading area and friction of SSL systems.

## Methods

**FIGURE 1:**
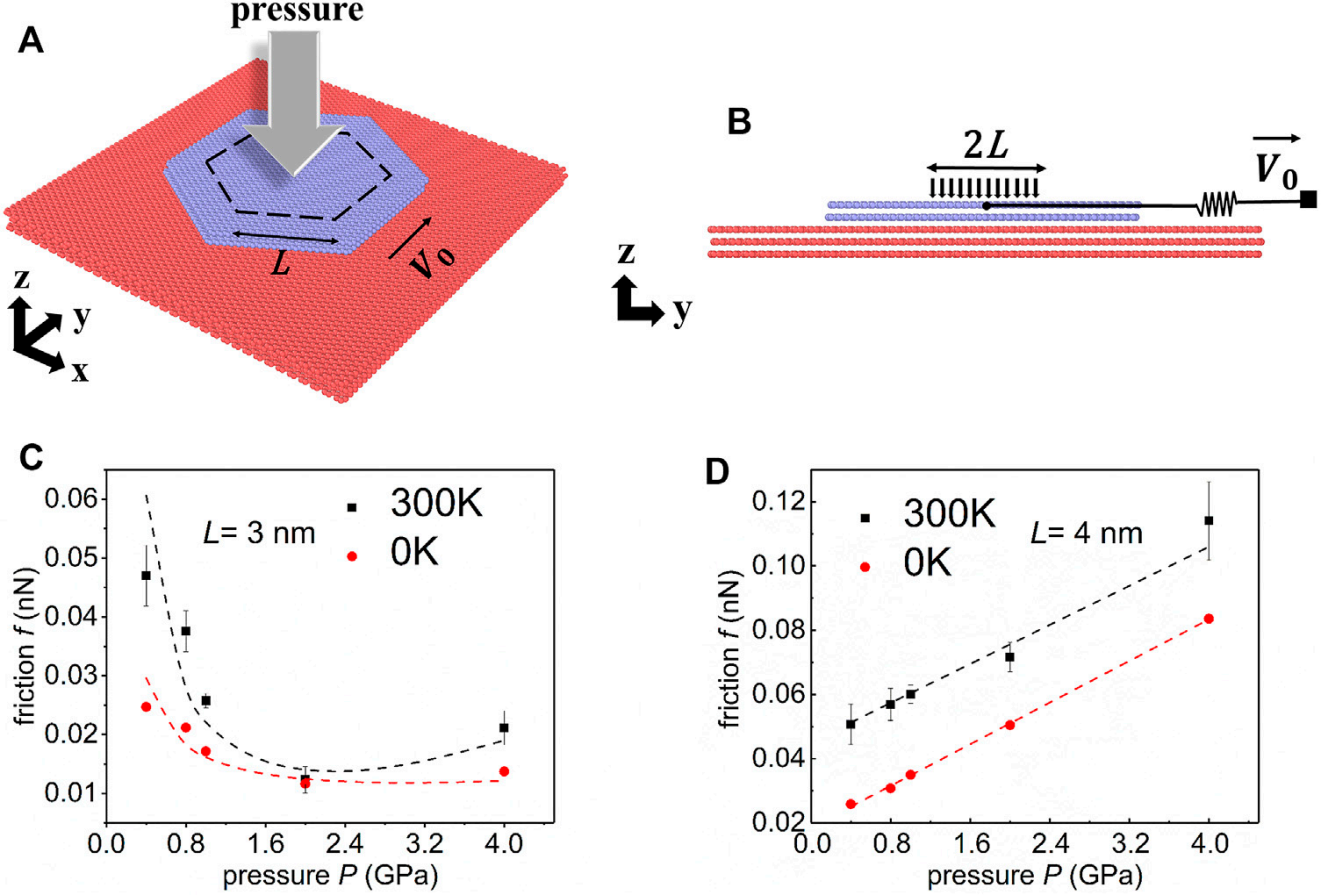
Simulation model and main results. **(A)** Schematic sketch of the simulation model. A hexagonal graphene flake (purple) on the strained graphene substrate (red). The area enclosed by the dashed hexagon is the loading area. *L* is the side length of the loading area of the hexagon. **(B)** Side view of the simulation model. **(C–D)** Dependence between the friction force *f* and the loading pressure *P* for **(C)** the small loading area and **(D)** the large loading area. The zero temperature results and room temperature results are shown in red and black respectively. The dashed curves are fitted with hook functions and linear functions.

As shown in Figures 1A,B, we choose a model consisting of five layers of graphene. The lower three layers are considered as the substrate (7,888 atoms each layer with the size 15.0 nm × 14.9 nm). The upper two layers are hexagonal flakes (2,400 atoms each layer) with the side length of 5 nm. The bottom layer is fixed to be a rigid body while the other layers are deformable. The misfit angle between the flake and the substrate is fixed to be 0°. Thus, to achieve a robust superlubric state, 4% in-plane biaxial stretching strains are applied to the substrate (Wang et al., 2019b; Wang et al., 2019c). Periodic boundary conditions are applied to the *x* and *y*-direction.

The hexagonal loading area enclosed by a black dashed line (Figure 1A) is concentric with the topmost graphene flake. The side length of the loading area is *L*. Within this area, a uniform normal force is applied to each atom. We calculate the normal pressure (for short, pressure) by dividing the normal force by the loading area. Two typical values of *L* are firstly chosen in our simulations: *L* = 3 nm corresponds to the small loading area, and *L* = 4 nm represents the large loading area. Notice here again that the side length of the flake is 5 nm. The pressure in the simulations ranges from 0.4 to 4 GPa to prevent damage to graphene (Mao et al., 2003; Guo et al., 2004).

The molecular dynamics simulations are performed using the LAMMPS package (Plimpton, 1995). The interlayer interaction is described by Lennard-Jones potential (Girifalco et al., 2000). Tersoff potential is adopted to describe the intralayer C–C bond interaction (Lindsay and Broido, 2010). A spring with the spring constant being *K*_s_ = 10 N/m is coupled to the center of mass of the topmost layer, and the other end of the spring moves with a constant velocity *V*_0_ = 10 m/s along + *y-*direction. In the simulation, we restrict the translational motion of the topmost flake along the *x-*direction. Along *x*-direction, springs are added to each carbon atom within the topmost layer of the graphite flake with spring constant $k=K_{s}/N_{top}$ to stand for the constraint exerted by the AFM tip, where $N_{top}$ is the total number of atoms of the topmost flake. The middle layer of the substrate is used as a buffer layer with Langevin thermostat applied to it. The normal load is applied directly to the topmost flake atoms. For all simulations, the timestep is fixed to be 1 fs. The friction force between the flake and the substrate is calculated by averaging the instantaneous resistance along the *y*-direction over at least 1 ns simulation time.

## Results

Figures 1C,D show the dependence between the friction *f* and the pressure *P* for the small and large loading area respectively. It is worth pointing out that for small loading area cases, friction shows a non-monotonic variation with the normal load, while a linear dependence is observed for large loading area cases. The variation trend does not change when the ILP potential is adopted to describe the interlayer interaction (see Supplementary Section S1 for more details).

Considering first the result for small loading area cases (*L* = 3 nm), we find that the friction decreases by ∼55% as the pressure increases from 0.4 to 2 GPa for zero temperature (red point). Then, as the pressure builds up and exceeds the transition pressure ∼2 GPa, the friction increases with the pressure. Defining the kinetic friction coefficient here by $\mu_{\text{k}}=\frac{\text{d}f}{A\text{d}P}$ (Liu et al., 2018b; Song et al., 2018), we find that $\mu_{\text{k}}$ in the simulations ranges from $-3.5\times 10^{-4}$ to $5.6\times 10^{-5}$ , where *A* is the loading area. Even using the engineering definition of friction coefficient, the ratio of friction to load, *f*/*PA*, we get a maximum friction coefficient of 5.0 × 10^−3^ Thus, considering the engineering definition of SSL (Martin et al., 1993), this small loading area system is superlubric. For room temperature, the kinetic friction reduces by 70% as the pressure increases from 0.4 to 2 GPa. Although the absolute values of friction are different at different temperatures, the non-monotonic characteristic between friction and pressure is similar. Based on the above observations, we can approximate the nonmonotonic behavior between friction force *f* and pressure *P* to the following hook function:

$$\begin{array}{c} f=\mathrm{kPA}+\frac{\Delta }{\mathrm{PA}}+f_{a} \end{array}$$ (1)

where k is estimated by fitting the curve, $f_{a}$ represents the offset friction force when the applied pressure is 0 induced by adhesion (Liu et al., 2017; Liu et al., 2018b; Liu et al., 2020b), and Δ is a fitting parameter. Specifically, friction scales linearly with the pressure when Δ =0 , which corresponds to the larger loading area cases. Δ appears when the applied pressure is not 0 and it represents the nonlinear behavior of negative correlation between friction and the pressure, which has also been observed in previous hBN/graphene heterojunction systems with small lattice mismatch (Mandelli et al., 2019). Fitting the results of the smaller loading area at 0 K with respect to Eq. 1, we get $k=4.28\times 10^{-5}$ , $\Delta =0.22nN^{2}$ , $f_{a}=0.0058\mathrm{nN}$ .

For the large loading area (*L* = 4 nm) at zero temperature, Δ=0 . In this case, $\mu_{k}$ and k have the same value. The slope (*k*) fitted by the least square method is $3.90\times 10^{-4}$ , which and indicates its superlubric nature. In addition, $f_{a}$ fitted at 0 K is 0.0186 nN. We also simulate the case with zero load and the result is 0.0193 nN, with a difference of only 3%.

Simulations performed at room temperature (black points in Figure 1D) yield the same trend and friction coefficient is fitted to be $4.2\times 10^{-4}.$ The similar linear dependence obtained at zero and room temperatures suggests the same physical mechanism behind. In addition, the above comparisons show that the correlation of friction on temperature is decoupled from the dependence between friction and normal loads.

## Discussion

**FIGURE 2:**
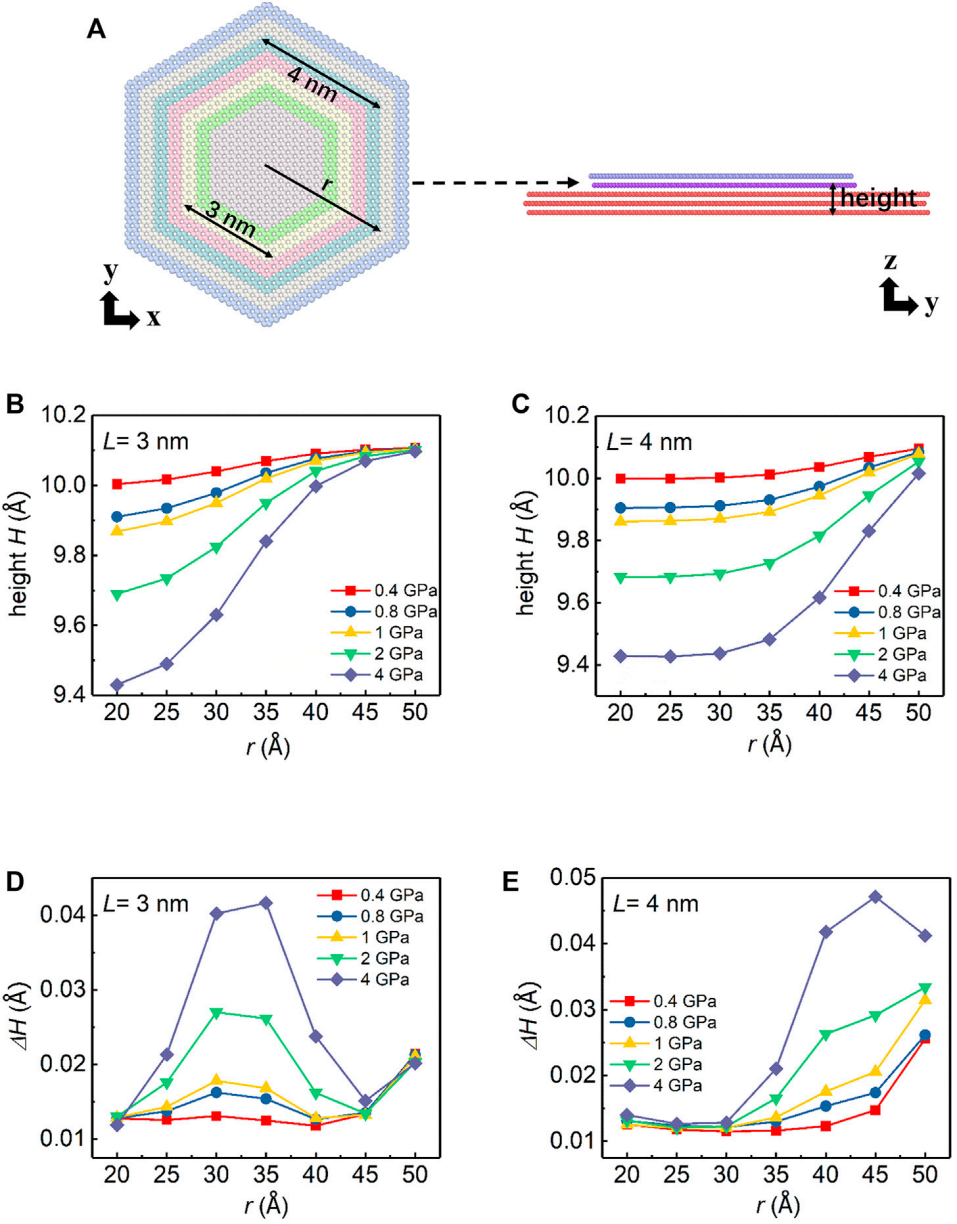
**(A)** Schematic sketch of the position of atoms on the graphene flake (the bottom layer of the graphite flake as indicated by the dashed arrow) with different colors. **(B)** Side view of the simulation model illustrating the layer we focus and its height *H*. **(C–D)** Spatial distribution of the height *H* shown in Panel **(B)** vs. loading pressure with different *L*. **(E–F)** Spatial distribution of the standard deviation of the height ΔH vs. loading pressure for different *L*.

To understand the load dependence of friction for different loading area cases, we analyze the spatial distribution of the average height *H* and the amplitude of the out-of-plane fluctuation ΔH of the atoms in different regions (Figures 2A,B) of the bottom layer of the graphite flake which is in contact with the substrate interfacial flake at 0 K.

As shown in Figures 2C,D, for both loading area cases, *H* increases from the center to the edge. However, the radial variation trend of the height varies. For the small loading area case, *H*(*r*) is a downward convex function inside the loading edge and follows up with an upward convex function outside the loading edge, where *r* denotes the radius of the circumscribed circle of the hexagon in which the atom is located (Figure 2A). For the large loading area case, *H*(*r*) is characterized by a uniformly downward convex function and *H* increases superlinearly from inside to outside. The difference between the two trends becomes even more prominent as the pressure increases. These height profiles, especially the profile containing an inflection point of the small loading area system, suggest an interplay among the normal load, the loading edge, and the flake edge.

The out-of-plane fluctuation ΔH (Figures 2E,F) provides more information to help us understand this interplay. The out-of-plane fluctuation of the flake is recognized to be the key for energy dissipation in superlubric systems (Van Wijk et al., 2014; Song et al., 2018; Liao et al., 2021). In the case of the small loading area, there are two peaks in ΔH(r) . One locates at the flake edge, and the other locates at the loading edge. For the large loading area cases, two peaks are almost overlapped since the edge of the loading area is close to the edge of the flake. Recent studies show that the dissipation behavior of edge atoms contributes greatly to friction, i.e., the edge effect. The edge atoms have a larger degree of freedom (Liao et al., 2021) and contribute 2–5 orders of magnitude greater friction dissipation than that of inner atoms (Wang et al., 2019a; Qu et al., 2020). Since the edge effect directly determines the friction of superlubricity, it is necessary to carefully understand the coupling between the loading edge and the flake edge.

For the small loading area case (Figure 2E), ΔH of the loading edge increases significantly with the increase of pressure. By contrast, there is only a marginal increase in ΔH of the flake edge. The observations suggest that for the small loading area case, the normal load hardly affects the atoms outside the loading edge. In other words, the dissipation from the edge effect is decoupled from the normal load.

For the large loading area case, two edges are nearly overlapped, which results in the coupling between the normal load and the edge effect. As we can see from Figure 2F, the edge has larger out-of-plane fluctuation as the normal load increases.

### Analysis About the Mechanisms

To better understand the energy dissipation route in our study, we analyze the frictional power (*p*_friction_) dissipated at zero temperature for all atoms in the second layer of the substrate, which is used as a buffer layer with Langevin thermostat. The dissipation power can be evaluated as follows: (Weiss and Elmer, 1997)

$$\begin{array}{c} p_{\mathrm{friction}}=\underset{i,\alpha =x,y,z}{\sum }m_{i}\eta_{\alpha }\langle {\left(v_{i,\alpha }-v_{\alpha ,\mathrm{com}}\right)}^{2}\rangle \end{array}$$ (2)

where $m_{i}$ denotes the mass of the *i*-th atom and $v_{i,\alpha }$ , $v_{\alpha ,\mathrm{com}}$ denotes the velocity of the *i*-th atom and the velocity of the center of mass of the flake along the α direction respectively, α=x,y,z . Here, $\eta_{\alpha }$ is the damping coefficient along the α direction and $\eta_{\alpha }=10{\text{ ps}}^{-1}$ for α=x,y,z . 〈…〉 denotes the ensemble average.

**FIGURE 3:**
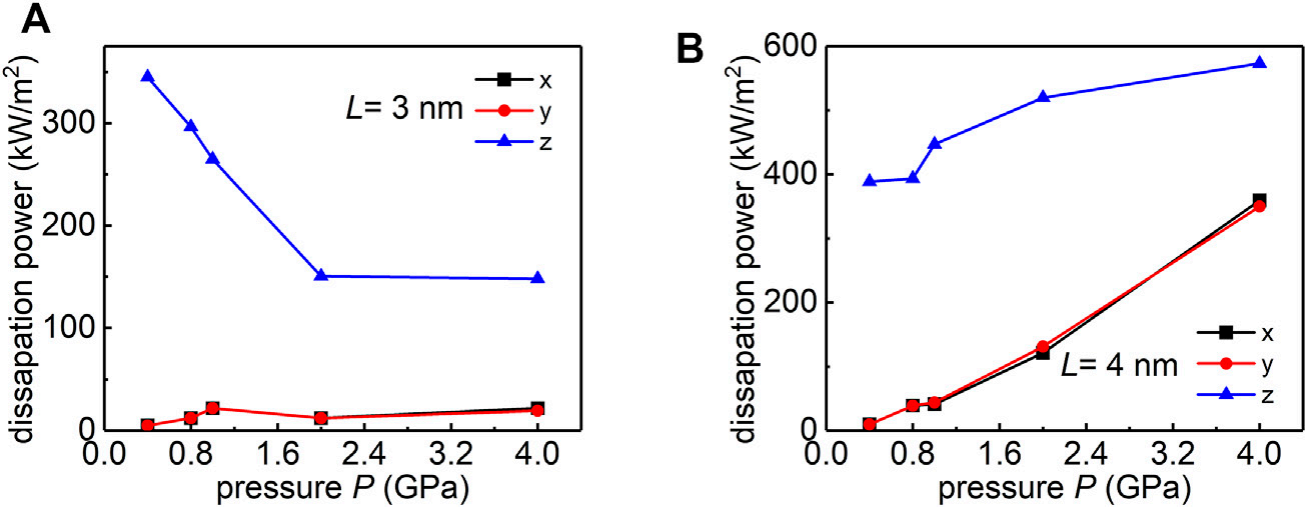
Load dependence of the frictional power dissipated at zero temperature for **(A)** small loading area case (*L* = 3 nm), and **(B)** large loading area case (*L* = 4 nm).

From Figures 3A,B, we observe that the dissipation power is dominated by the *z* component (blue curve), which is in consistent with previous reports on superlubric contacts (Song et al., 2018; Mandelli et al., 2019). For smaller loading areas cases (*L* = 3 nm), over ∼80% of the energy dissipation is accounted for the out-of-plane fluctuation. For large loading area cases (*L* = 4 nm), when the pressure increases from 2 to 4 GPa, both in-plane and out-of-plane dissipation increase with the normal load, and the in-plane dissipation becomes comparable to the out-of-plane dissipation. These analysis rationalize the linear dependence between the friction and normal load in large loading area cases.

Based on the above findings, we propose an analytic model to quantitatively understand the dependence of friction on pressure influenced by the size of the loading area. The hexagonal flake is divided into two areas: the loading area and the free area. The loading area refers to the hexagonal area which is concentric with the interfacial flake enclosed by a black dashed line of side length L, while the free area refers to the rest area of the flake. (Supplementary Figure S2 in supplementary Section 2). In the free area, the per-atom friction force is $f_{0}$ . From our data fitting (details in supplementary Section 3), $f_{0}$ is estimated to be $7.75\times 10^{-6}\text{ nN}$ . Within the loading area, the per-atom friction is $f_{\text{N}}$ . Thus, the total friction can be expressed as

$$\begin{array}{c} f=N_{0}f_{0}+\left(N-N_{0}\right)f_{N} \end{array}$$ (3)

where $N_{0}$ denotes the number of atoms in the free area and *N* is the total atom number of the interfacial layer.

Then, according to Eqs.1, 3, we have: $f_{N}=k^{\prime }PA_{0}^{\prime }+\frac{\Delta^{\prime }}{PA_{0}^{\prime }}+f_{a}^{\prime }$ , where ${A^{\prime }}_{0}$ is the area of one graphene carbon atom, $\mu^{\prime }$ , $\Delta^{\prime }$ , $f_{a}^{\prime }$ are parameters to be fitted (details in supplementary Secion 3). When the loading area is small (L≤3nm) , $k^{\prime }=4.28\times 10^{-5}$ , $\Delta^{\prime }=2.92\times 10^{-7}\text{nN}\cdot \text{nN}$ , $f_{a}^{\prime }=-7.648\times 10^{-4}\text{nN}$ . When the loading area is large, $\Delta^{\prime }\to 0$ as we discussed previously, $k^{\prime }=3.90\times 10^{-4}$ , $f_{a}^{\prime }=f_{0}$ .

### Discussion About the Model

**FIGURE 4:**
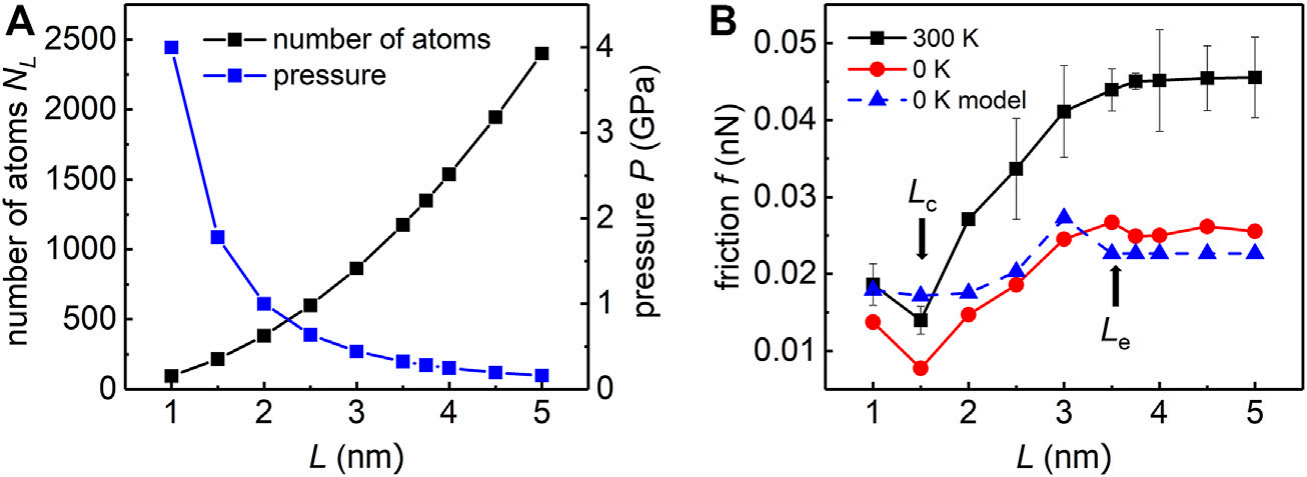
Simulation for applying the same total force in the loading area. **(A)** Numbers of atoms in the loading area *N*_L_ and pressure versus *L*. The product of *N*_*L*_ and pressure remains constant. **(B)** Friction force of the flake along y direction versus *L* at 0 K (red) or 300 K (black). The result obtained by our model at 0 K is shown in blue.

In order to build up the bridge between our simulation results and realistic experimental measurements, and also verify the applicability of above theoretical model, we perform additional simulations with similar set-ups as shown in Figure 1A. Instead of using the same pressure in two different loading area cases in previous simulations, here we keep the total normal force as a constant for different loading area cases. In other words, the normal pressure decreases as the loading area increases. By choosing the total force as $F_{\text{N}}=10.33nN$ , the number of atoms in the loading area and pressure for different *L* is shown in Figure 4A. Specifically, for *L* = 2 nm, the number of atoms in the loading area is $N_{\text{L}}=N-N_{0}$ is 384 and the corresponding pressure is 1 GPa. While for *L =* 2.5 nm, $N_{\text{L}}$ is 600 with the pressure 0.64 GPa. In our simulations, the minimum pressure (160 MPa) is achieved when all flake atoms experience a uniformly distributed normal load. And the pressure reaches its maximum (∼4 GPa) when *L* = 1 nm. Note that even this maximum normal pressure is below the load to cause structural distortion in the graphene (Mao et al., 2003; Guo et al., 2004).

With this new simulation set-ups, we study the dependence between the friction *f* and the size of the loading area *L*. We find a transition size of friction with different trends of the loading area, $\;L_{\text{e}}$ , which is between 3 and 4 nm. So far, we obtain the value of $L_{\text{e}}$ by simulation results. As shown in Figure 4B, when the side length of the loading area becomes greater than the transition size ( $L>L_{\text{e}}$ ), the friction force remains constant and does not correlate with *L*. For this case, the loading edge and the flake edge effectively overlaps, which further causes the coupling between the loading and the edge effect. For cases that $L\leq L_{\text{e}}$ , the friction force decreases/increases as the size of the loading area decreases for $L>L_{c}$ or $L<L_{c}$ , where $L_{\text{c}}$ is a turning point (∼1.5 nm) derived from the model we proposed above (see Supplementary Section S6 for more details).

To be specific, the friction decreases up to ∼68% at 0 K and friction decreases up to ∼66% at 300 K when *L* decreases from 3 to 1.5 nm, which indicates that reducing the loading area could be a promising way to effectively reduce the friction for the superlubric contacts. For 0 K case, the above theoretical model successfully predicts two transition sizes $L_{\text{c}}$ and $L_{\text{e}}$ (see Supplementary Section S6 for more details). In addition, based on the model, the magnitude and the variation trend of the estimated friction are quantitatively consistent with the simulations, which further illustrates the rationality and accuracy of the theoretical model.

To fully explain the friction dependence discovered here, we also explore the influences from other characteristic lengths of the system, including the moiré size and the flake size, and try to extract some dimensionless invariants (see Supplementary Sections S4–5 for more details). However, it seems that the friction dependence is non-trivial, and it does not explicitly depend on these physical quantities. At the present stage, it seems difficult to find some physical quantities to fully describe this dependence.

## Conclusion

In summary, by studying the normal load dependence of friction in the structural superlubric system with extensive MD simulations, we discover two different dependences for the same simulation model: a non-monotonic dependence and a textbook linear dependence. The main reason for this difference lies in the size of the loading area. For small loading area cases, the dependence between the friction and normal load is non-monotonic and can be approximated by a hook function. For large loading area cases, the friction is proportional to the normal load. Analysis on the structure and energy dissipations shows that the friction dissipation from the flake edge is significantly affected by the normal load for large loading area cases, while the friction dissipation from the flake edge of small loading area cases is hardly affected by the normal load. The essence behind these observations stems from the coupling between the normal load and the edge effect of SSL systems. Besides, we find that by further reducing the loading area, the friction can be reduced by more than 65% compared to the larger loading area cases, providing a new way to effectively reduce and control friction. Our discoveries suggest that in order to achieve negative correlation between friction and normal load experimentally, 1) the contact should be superlubric, and 2) the loading area should be small enough to eliminate the coupling between the load and the edge effect. Given that the existing AFM-based experiments could meet these two requirements (Wang et al., 2015; Vu et al., 2016; Wang et al., 2019d), we look forward to experimental verification of our findings in the near future. Due to the similarity of different 2D materials in crystallography and mechanics (Geim and Grigorieva, 2013; Novoselov et al., 2016), our findings may apply to other superlubric 2D materials, such as graphene/hBN and graphene/MoS_2_.

## Data Availability

The raw data supporting the conclusion of this article will be made available by the authors, without undue reservation.
