# Supplementary material for: Negative or Positive? Loading Area Dependent Correlation Between Friction and Normal Load in Structural Superlubricity
Source: Front Chem. 2022 Feb 1;9:807630. doi: 10.3389/fchem.2021.807630 (PMC8844525; doi:10.3389/fchem.2021.807630)
Supplement: Supplementary file 1 [file DataSheet1.docx]

Negative or positive? Loading area dependent correlation between friction and normal load in structural superlubricity

Supplementary Materials

1. Details for the simulations of ILP potential

2. Figures for the loading areas and the free areas

3. Details for the derivation of $f_{0}$ and $f_{N}$

4 Details for the simulation to explore the friction dependence on the side length of moiré pattern

5. Details for the simulation to explore the friction dependence on the ratio between the side length of the loading area and the flake size

6. Details for the derivation to explain the trend in Section 6

1. **Details for the simulations of ILP potential**

We perform additional simulations with registry-dependent interlayer (ILP) potential(Ouyang et al., 2020). For these ILP-based simulations, we delete the rigid bottom layer in the substrate to improve the computational efficiency. At the same time, to keep the accuracy of the simulation, we tethered *z*-directional springs to the carbon atom in the second layer to reproduce its interaction with the (deleted) rigid bottom layer. The spring constant *k_z_* equals 2.7 N m^−1^, which is derived from the interaction of two adjacent graphene layers with harmonic approximation(Guo et al., 2012). Thus, the two lower layers are considered as the substrate (5076 atoms each layer with the size 12.0 nm × 11.9 nm). (Figure S1A, B)

We find that the qualitative results don’t change. For small loading area cases (*L* =3 nm), friction shows negative correlation between friction and the pressure, while a positive correlation is observed for large loading area cases (*L* =4 nm).(Figure S1C, D)


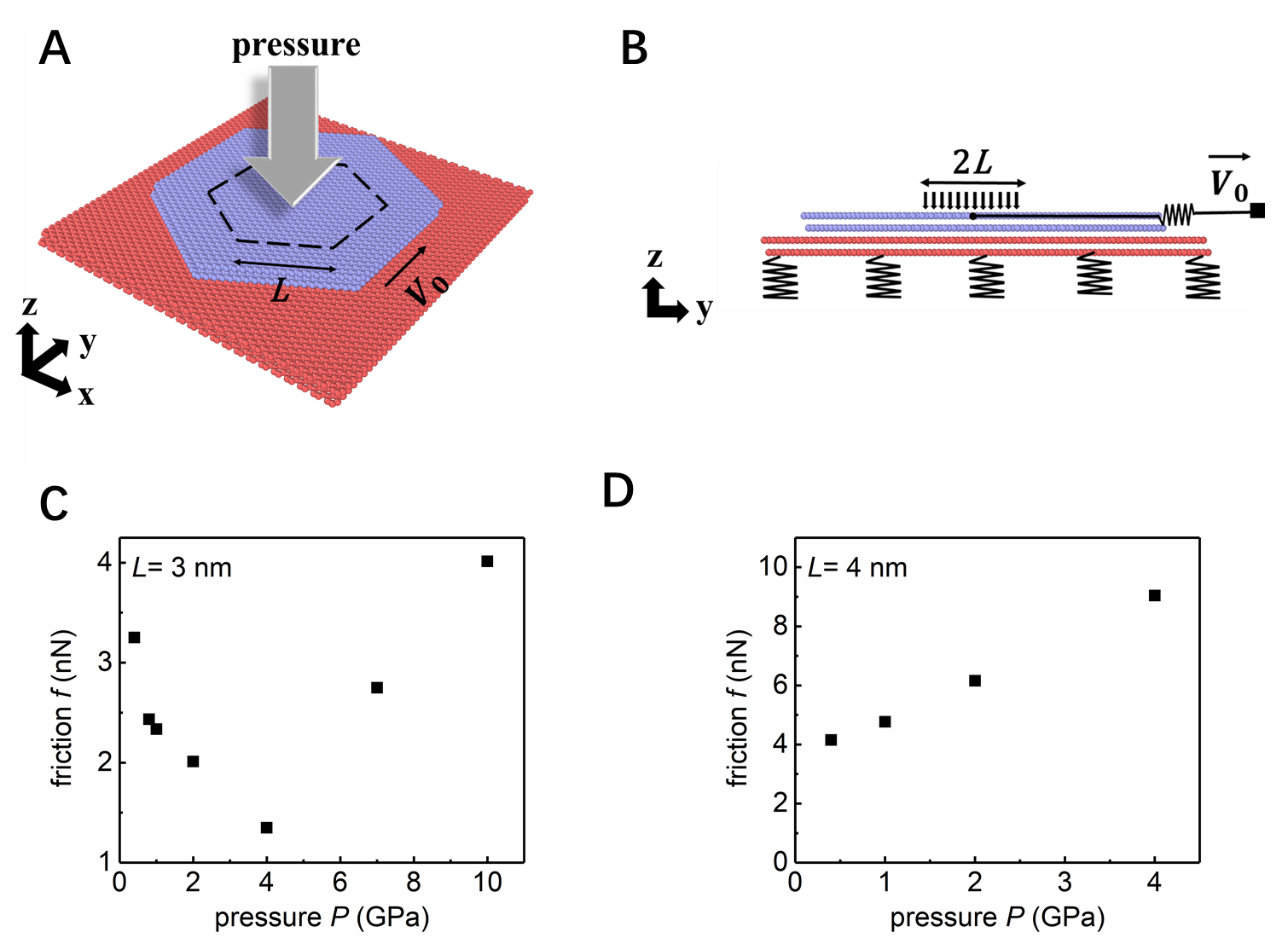


**FIGURE S1**. ILP-based simulation model and results. (A) Schematic sketch of the simulation model. A hexagonal graphene flake (purple) on the strained graphene substrate (red). The area enclosed by the dashed hexagon is the loading area. *L* is the side length of the hexagonal loading area. (B) Side view of the simulation model and set-ups. (C-D) Dependence between the friction force *f* and the loading pressure *P* for (C) the small loading area and (D) the large loading area.

1. **Figures for the loading areas and the free areas**


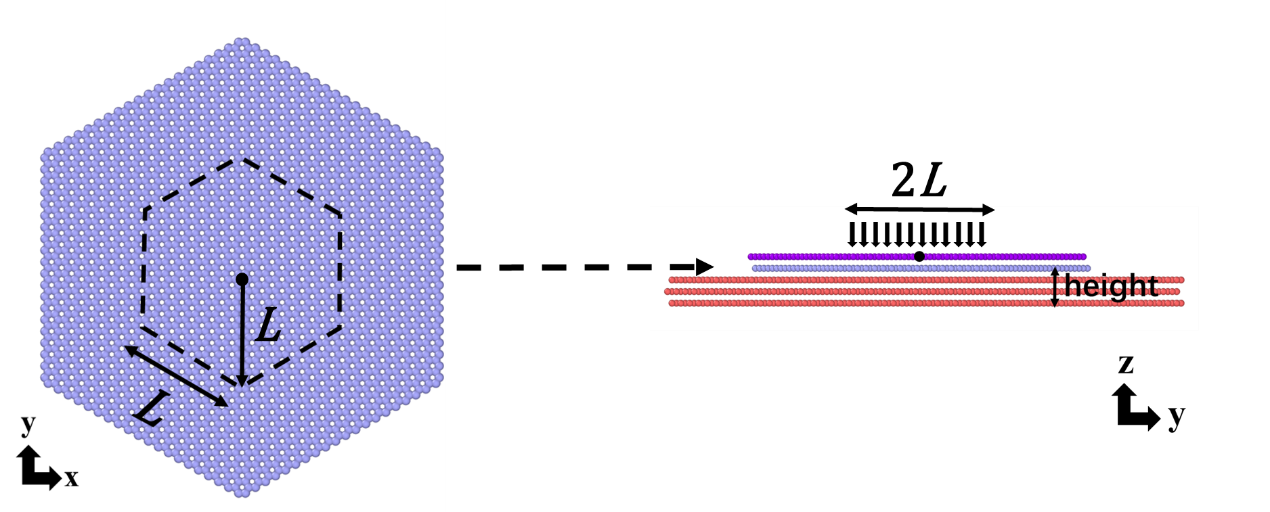


**FIGURE S2**. Schematic sketch of different areas on the graphene flake (the bottom layer of the graphite flake as indicated by the dashed arrow). The loaded area enclosed by the dashed hexagon is the loading area. *L* is the side length of the loading area of the hexagon.The rest of the flake is the free area.

1. **Details for the derivation of** $\boldsymbol{f}_{\mathbf{0}}$ **and** $\boldsymbol{f}_{\boldsymbol{N}}$

The intercept value($f_{a}$) of linearly fitted at 0 K in Fig. 1D is 0.0186 nN, which denotes the fitting value of friction when the flake is not loaded. The average friction force per atom in the free area $f_{0}=f_{a}/N=7.75\times{10}^{-3}$ nN, where *N* denotes the total number of the atoms.

Within the loading area, the per-atom friction is $f_{N}$. Thus, The total friction can be expressed as

$$\begin{aligned} \boldsymbol{f=}\boldsymbol{N}_{\boldsymbol{0}}\boldsymbol{f}_{\boldsymbol{0}}\boldsymbol{+}\left( \boldsymbol{N-N}_{\boldsymbol{0}} \right)\boldsymbol{f}_{\mathbf{N}}\boldsymbol{\#}\left( S1 \right) \end{aligned}$$

where $N_{0}$ denotes the number of atoms in the unloaded area and *N* is the total atom number of the interfacial layer.

According to previous fitting (**Figure 1C, D**) and Eq. (S1), we have:

$$\begin{aligned} \boldsymbol{f}_{\boldsymbol{N}}\boldsymbol{=}\frac{\left( \boldsymbol{f-}\boldsymbol{N}_{\boldsymbol{0}}\boldsymbol{f}_{\boldsymbol{0}} \right)}{\boldsymbol{N-}\boldsymbol{N}_{\boldsymbol{0}}}\boldsymbol{=}\frac{\boldsymbol{kPA+}\frac{\boldsymbol{\Delta}}{\boldsymbol{PA}}}{\boldsymbol{N-}\boldsymbol{N}_{\boldsymbol{0}}}\boldsymbol{+}\left( \frac{\boldsymbol{f}_{\boldsymbol{a}}}{\boldsymbol{N-}\boldsymbol{N}_{\boldsymbol{0}}}\boldsymbol{-}\frac{\boldsymbol{N}_{\boldsymbol{0}}\boldsymbol{f}_{\boldsymbol{0}}}{\boldsymbol{N-}\boldsymbol{N}_{\boldsymbol{0}}} \right)\#\left( S2 \right) \end{aligned}$$

Therefore, $k^{'}=\frac{kA}{(N-N_{0}){A_{0}}^{'}}$,$\Delta^{'}$=$\frac{\Delta{A_{0}}^{'}}{A(N-N_{0})}$=$\frac{\Delta}{\left( N-N_{0} \right)^{2}}$, $f_{a}^{'}=\left( \frac{f_{a}}{N-N_{0}}-\frac{N_{0}f_{0}}{N-N_{0}} \right)$,where $A_{0}'$ is the area of an carbon atom.

1. **Details for the simulation to explore the friction dependence on the side length of moiré pattern**

Firstly, based on our existing simulations, we compare the value of $L_{e}$ and the side length of moiré pattern. The value of $L_{e}$ (Fig. 4B) (~3 nm) is smaller than the side length of moiré pattern $L_{moiré}$ (~3.75 nm).

In order to further explore the correlation between $L_{e}$and $L_{moiré}$, we perform additional simulations to study the relationship between friction and different *L* around $L_{e}$. We apply 2% in-plane biaxial stretching strains to the substrate. (5076 atoms each layer with the size 12.0 nm × 11.9 nm) The rest of the settings are the same as the Section 6 of the main text. For this new model, $L_{moiré}$ is 7.34 nm, which is larger than the side length of the flake (5 nm).

Simulation results are shown in Fig S3. We find a transition size$L_{e}$ between 3 to 4 nm, which agrees with our previous results in the manuscript: When *L*>$L_{e}$, the friction force remains constant and does not correlate to *L*; When *L*$\leq L_{e}$, the friction force decreases as the size of the loading area. From the above results, we did not find a trivial relation between the moiré size and the transition size $L_{e}$.





**FIGURE S3**. Friction force of the flake along *y*-direction versus *L* at 0 K.

1. **Details for the simulation to explore the friction dependence on the ratio between the side length of the loading area and the flake size**

We apply a larger in-plane strain ($\epsilon$=7%) to the substrate (9088 atoms each layer with the size 17.4 nm × 17.3 nm) . Firstly, we use a larger graphene flake with the side length of 7.5 nm. Besides, we use three different side lengths of the loading area: 4.5 nm, 6 nm, and 7.5 nm. The rest of the settings are the same as the Section 2 in the manuscript at 0K.

In our manuscript, the ratio between the size of the normal loading area *L* and the flake size *L*_flake_ is 0.6 and 0.8 for the small loading area and the large loading area correspondingly. This corresponds to the simulations with *L*= 4.5/6 nm.

For *L*= 4.5/6 nm, friction shows a non-monotonic variation with the normal load, while a linear dependence is observed for *L*= 7.5 nm (Figure S6). For the case with the ratio equal to 0.8, friction shows the same non-monotonic variation with the normal load as the ratio equal to 0.6. It seems that the dependence between the friction and loading area is non-trivial, and it does not explicitly depend on the ratio between the loading area size and the flake size.


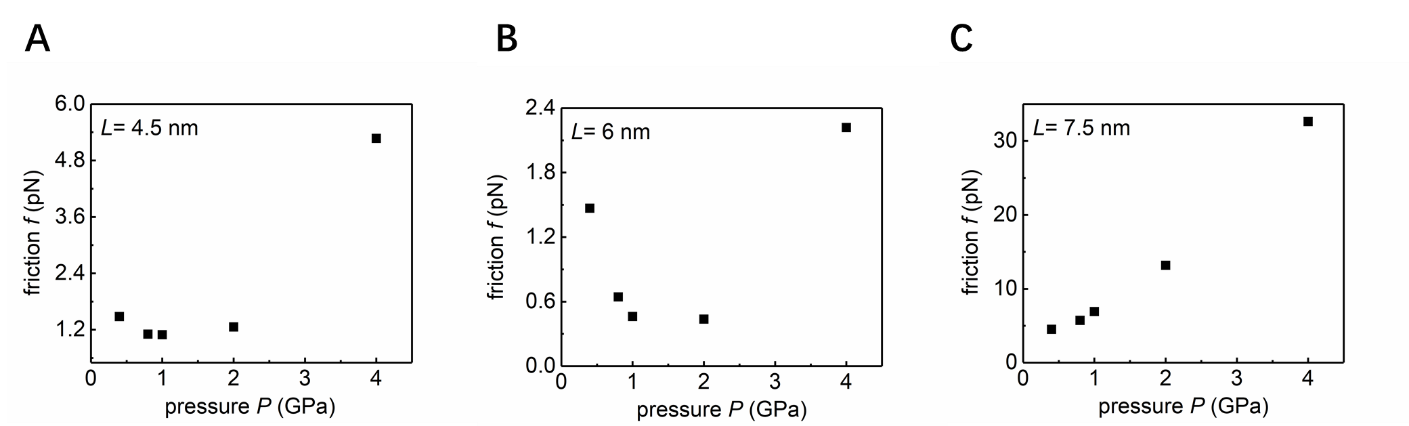


**FIGURE S4**. Dependence between the friction force *f* and the loading pressure *P* for (A) *L*= 4.5 nm (B) *L*= 6 nm (C) *L* = 7.5 nm.

1. **Details for the derivation to explain the trend in Section 6**

This trend for the simulations in Section 6 can be well explained by our model. The total force applied is $F_{N}$=10.33 nN. $F_{N}$ is also equal to $C=\frac{\left( N-N_{0} \right)P}{\rho}$, where $\rho$ is the number of atoms per unit area , *P* is the pressure and C denotes a constant.

According to previous results, we have:

$$\begin{aligned} \boldsymbol{f}_{\boldsymbol{N}}\boldsymbol{=}\boldsymbol{k}^{\boldsymbol{'}}\boldsymbol{P}\boldsymbol{A}_{\boldsymbol{0}}\boldsymbol{'+}\frac{\boldsymbol{\Delta}^{\boldsymbol{'}}}{\boldsymbol{P}{\boldsymbol{A}_{\boldsymbol{0}}}^{\boldsymbol{'}}}\boldsymbol{+}\boldsymbol{f}_{\boldsymbol{a}}^{\boldsymbol{'}}\#\left( S3 \right) \end{aligned}$$

We discuss the trend in Section 6 in two cases:$L\leq L_{e}$ and $L>L_{e}$:

For the case of $L\leq L_{e}$, we use the parameters for the smaller laoding areas: $k'=4.28\times{10}^{-5}$,$\Delta^{'}=2.92\times{10}^{-7} \mathrm{nN}\cdot nN$, ${f_{a}}^{'}=-7.648\times{10}^{-4}\mathrm{nN}$. We derive the followings combined with (S1) and (S3):$f=\left( {N-N}_{0} \right)^{2}\frac{\Delta^{'}}{c^{'}} - ({N-N}_{0}){(f}_{0}-f_{a}')+ k'c' + N_{0}f_{0}$, where $c^{'}=C\rho A_{0}'$. The number of atoms in the loaded area ${N-N}_{0}$ and *L* have the following geometric relationship: $({N-N}_{0})$ *=*${6(L/L_{0})}^{2}$,$L_{0}=0.25 \mathrm{nm}$*.* When $L_{\min}=\left( \frac{c'{(f}_{0}-{f_{a}}^{'})}{12\Delta^{'}} \right)^{1/2}$, $f$ reaches to a minimum. The value of $L_{\min}$ calculated by previous fitted results is approximate to $L_{c}(\sim1.5 \mathrm{nm})$, which we obtained in our simulations. When $L\leq L_{\min}$, friction decreases as *L* increases while friction increases as *L* increases when $L_{\min}<L\leq L_{e}$.

For the case of $L>L_{e}$, $\Delta^{'}$ is zero as we discussed previously, $k^{'}=3.90\times{10}^{-4}$, ${f_{a}}^{'}=$ $f_{0}$. We derive the following formula combined with Eq. (S1) and Eq. (S3): $f$*=*$k'c'$+$Nf_{0}$. All of these are constants in our case so the friction remains constant.

Reference

GUO, Z., CHANG, T., GUO, X. & GAO, H. 2012. Mechanics of thermophoretic and thermally induced edge forces in carbon nanotube nanodevices. *Journal Of the Mechanics And Physics Of Solids,* 60**,** 1676-1687.

OUYANG, W., AZURI, I., MANDELLI, D., TKATCHENKO, A., KRONIK, L., URBAKH, M. & HOD, O. 2020. Mechanical and Tribological Properties of Layered Materials under High Pressure: Assessing the Importance of Many-Body Dispersion Effects. *Journal Of Chemical Theory And Computation,* 16**,** 666-676.
